# Assessing the Interpretation of Molecular Test Results in the Diagnosis of Bloodstream Infections

**DOI:** 10.3390/diagnostics14090915

**Published:** 2024-04-27

**Authors:** Natalia Słabisz, Patrycja Leśnik, Katarzyna Żybura-Wszoła, Ruth Dudek-Wicher, Urszula Nawrot, Jacek Majda

**Affiliations:** 1 Department of Laboratory Diagnostic, 4th Military Clinical Hospital in Wroclaw, 53-114 Wroclaw, Poland; kzybura@4wsk.pl (K.Ż.-W.); jmajda@4wsk.pl (J.M.); 2 Department of Microbiology, Faculty of Medicine, Wroclaw Medical University, 50-386 Wroclaw, Poland; patrycja.lesnik@gmail.com; 3 Department of Pharmaceutical Microbiology and Parasitology, Faculty of Pharmacy, Wroclaw Medical University, 50-367 Wroclaw, Poland; r.dudek.wicher@gmail.com (R.D.-W.); urszula.nawrot@umw.edu.pl (U.N.); 4 Department of Preclinical Sciences, Pharmacology and Medical Diagnostics, Faculty of Medicine, Wroclaw University of Science and Technology, 58-376 Wroclaw, Poland

**Keywords:** molecular diagnostic, bloodstream infections, sepsis, antimicrobial stewardship program, antibiotics

## Abstract

A retrospective study at the 4th Military Clinical Hospital in Wroclaw, Poland, assessed PCR testing alongside blood cultures to guide antimicrobial therapy decisions in hospitalized patients, to determine how much time the results of the molecular tests preceded conventional methods. Among 118 patients, *Staphylococcus aureus* (37%) and *Escherichia coli* (21%) were the most common bloodstream infection agents. Blood cultures utilized the BacT/ALERT 3D system, and molecular diagnostics were conducted using the FilmArray platform with the BIOFIRE BCID2 panel. Methicillin susceptibility was observed in 66% of *S. aureus* strains, while 26% of Gram-negative bacilli exhibited an ESBL phenotype. Therapeutic decisions based on molecular test results were often incorrect for *S. aureus* infections, particularly MSSA (64.5%), but generally accurate for Gram-negative bacilli. The median times from positive blood culture to BCID2 and pathogen identification/susceptibility were 10 h and 52 h, respectively. Molecular diagnostics facilitated faster initiation of appropriate antibiotic therapy, highlighting the need to educate medical staff on proper interpretation. Consulting within an antimicrobial stewardship program (ASP) could enhance the benefits of implementing molecular methods in bloodstream infection diagnostics.

## 1. Introduction

Systemic infections in the form of bacteremia, sepsis, or septic shock are associated with high mortality rates, ranging from 25–80%. It is estimated that there are approximately 30 million cases of bloodstream infections (BSIs) and approximately 6 million deaths worldwide, every year [1,2]. According to European data, 1.2 million BSIs are registered annually [3]. It should be highlighted that these statistics may be significantly underestimated, due to the frequent lack of official reporting of cases. Due to the lack of official reporting, the number of sepsis cases in Poland does not reflect the actual situation and is assessed based only on epidemiological data from other European countries [4,5]. The prognosis and the course of the disease depend on many factors. The patient’s age and comorbidities will constitute risk factors for a severe course of the infection, as will the virulence or antibiotic resistance of the microorganism itself [6]. Gram (+) bacteria cause more than 50% of all BSI cases; the main etiological factors include coagulase-negative staphylococci (CoNS), *Staphylococcus aureus*, and *Enterococcus* spp. [1]. On the other hand, at least 30% of BSIs are caused by Gram (−) microorganisms. These infections are associated with higher mortality rates (15–29%) related to the occurrence of antibiotic resistance [6]. The key element that increases the patient’s chances of survival is the quick implementation of appropriate antibiotic therapy. Incorrect therapeutic decisions are associated with a five-fold reduction in survival among patients with sepsis. In the era of constantly increasing microbial resistance, it seems rational to use broad-spectrum empirical antibiotic therapy, which, on the one hand, guarantees therapeutic success but, on the other hand, has consequences such as post-antibiotic diarrhea of *Clostridioides difficile* etiology or resistant strain selection [7,8,9]. Swift identification of the causative pathogens and potential resistance markers in blood cultures is essential for administering optimal therapy promptly, thereby enhancing patient survival. Research indicates that any delay in providing appropriate antimicrobial therapy, whether due to prolonged time for pathogen identification or antimicrobial resistance, leads to heightened mortality rates [10]. Conventional culture of microorganisms from blood using automatic systems remains the gold standard for diagnosing BSIs. However, the main disadvantage of this method is the long waiting time for the final result. Depending on the analyzers owned by the microbiological laboratory, the time from obtaining a positive blood culture to the identification and determination of drug resistance in the microorganism constituting the etiological agent may range from 48–168 h [9,11]. The current IDSA (Infectious Diseases Society of America) guidelines advocate for incorporating rapid diagnostic testing into a comprehensive antimicrobial stewardship program (ASP). Implementing molecular biology methods into the diagnostic scheme of BSIs significantly reduces the time needed to make optimal therapeutic decisions [12]. According to the Shah et al. study, the median time to molecular test results was 21 h from a positive blood culture. The time to pathogen identification using MALDI-ToF, but not directly from the blood culture, was 42 h. Susceptibility was performed using Vitek2 (bioMérieux, Marcy l’Etoile, France), and the median time to result was 49 h [13].

Systems using a positive blood culture for testing are often based on multiplex PCR (polymerase chain reaction) methods, which allow for the simultaneous detection of many species of microorganisms and resistance genes. The BCID2 (Blood Culture Identification Panel 2) (bioMérieux, Marcy l’Etoile, France) septic panel enables the detection of 33 pathogens (26 species/genera of bacteria, seven species of yeast-like fungi) associated with BSIs, as well as the detection of ten antibiotic resistance genes, determining, among others, production of carbapenemases, extended-spectrum β-lactamases (ESBL), or methicillin resistance in staphylococci [14,15,16]. The meta-analysis conducted by Peri et al. assessing the BCID2 performance for pathogen identification and resistance markers detection, compared to culture methods, remains the gold standard in diagnosing BSIs. The combined specificity of the assay was outstanding (>97%) across most investigated target subgroups. Additionally, the combined sensitivity was notably high for the primary determinants of bloodstream infection, including *Enterobacterales* (98.2%), *S. aureus* (96.0%), *Streptococcus* spp. (96.7%), *P. aeruginosa* (92.7%), and *E. faecalis* (92.3%), as well as *bla*_CTX-M_ (94.9%), carbapenemases (94.9%), and *mecA/C* and MREJ (93.9%). The potential inconsistency between genotypic and phenotypic resistance is a limitation of the BCID test’s applications. Situations can arise where, despite the presence of a resistance gene, it may not be expressed, resulting in the strain being phenotypically susceptible to a particular antibiotic. Conversely, the strain’s resistance may stem from different resistance genes than those detected in the BCID2 panel. However, there are situations in which obtaining a specific result should not raise doubts regarding further therapeutic decisions. An example of this could be the detection of *mecA/C* + MREJ in *Staphylococcus aureus*, where the association between the presence of the resistance gene and phenotypic resistance is clear and very well documented [16]. Therefore, many publications indicate that expensive molecular tests can bring tangible benefits, including financial ones, only when these results will be consulted within the team for the hospital antibiotic policy program and appropriate therapeutic decisions will be quickly implemented. The correct interpretation of PCR test results requires knowledge of bacterial genes that determine antibiotic resistance and the possibility of resistance resulting from mechanisms other than those genetically encoded. An essential role in the team for hospital antibiotic policy is played by a clinical microbiologist who, having the appropriate knowledge, can correctly assess the obtained result and communicate their interpretation to clinical teams [9,12,17,18].

The study aimed to assess the ability to interpret the results of PCR tests performed on positive blood cultures, based on therapeutic decisions made by clinicians, and the potential impact of antibiotic therapy management.

## 2. Materials and Methods

### 2.1. Study Design

The results of PCR tests from positive blood cultures and the disease history of adult patients hospitalized in 2021–2022 at the 4th Military Clinical Hospital, a 500-bed medical center in Wroclaw, Poland, were retrospectively assessed. The inclusion criteria were: age above 18 years, first episode of positive blood culture during hospitalization, and absence of consultation regarding the molecular test results within the hospital antimicrobial stewardship. The exclusion criteria were: positive blood culture was a control culture, patient’s demise before the identification, and antibiotic susceptibility test results from routine blood culture. The study’s retrospective nature will allow for the evaluation of molecular test interpretation assessments in relation to studies conducted in subsequent years. Such a comparison will enable an evaluation of the effectiveness of the molecular methods training conducted in the meantime for the diagnosis of bloodstream infections. Among patients enrolled in the study, blood culture was performed as part of routine microbiological diagnostics, and then, after obtaining a positive culture using automatic systems, the study was extended to include molecular diagnostics. Information on the empirical antibiotic therapy used and its possible changes after obtaining the PCR test result was obtained from the patient’s medical history in the electronic medical records (EMR) system. All analyzed results were not consulted within the hospital’s antimicrobial stewardship, and their interpretation and further therapeutic decisions were made only by the attending physician.

### 2.2. Blood Culture

Blood culture was performed in an automated BacT/ALERT 3D instrument system (bioMérieux, Marcy l’Etoile, France) at 37 °C as part of a 5-day incubation protocol. The patient’s blood was inoculated into culture media containing an antibiotic inactivator dedicated to the BacT/ALERT system (BacT/ALERT FN PLUS, BacT/ALERT FA PLUS (bioMérieux, Marcy l’Etoile, France). The positive blood cultures were Gram-stained, then streaked onto Columbia Agar (bioMérieux, Marcy l’Etoile, France), Chocolate agar (bioMérieux, Marcy l’Etoile, France), and MacConkey (bioMérieux, Marcy l’Etoile, France) for overnight incubation in 5% carbon dioxide at 37 °C. The VITEK-2 automated system (bioMérieux, Marcy l’Etoile, France) was used for isolates identification and antimicrobial susceptibility testing.

### 2.3. Pathogen Identification and Susceptibility Testing

The identification and susceptibility testing of microorganisms cultured from the blood cultures were performed using the Vitek2 system (bioMérieux, Marcy l’Etoile, France). The VITEK2 AST-N331 and AST-N332 panels were employed for testing the antibiotic susceptibility of Gram-negative bacilli, while AST-P643, AST-P644, and AST-ST01 were utilized for Gram-positive cocci. Additionally, AST-YS08 was utilized for fungi. The detection of carbapenemases was conducted using an immunochromatography test (RESIST-5 O.O.K.N.V, CorisBioConcept, Gembloux, Belgium). Susceptibility test results were interpreted based on the current criteria established by the European Committee on Antimicrobial Susceptibility Testing (EUCAST) [19].

### 2.4. Molecular Test

A molecular study was conducted on the FilmArray platform (bioMérieux, Marcy l’Etoile, France), using the BIOFIRE Blood Culture Identification Panel 2 (BCID2) (bioMérieux, Marcy l’Etoile, France) by the manufacturer’s instructions provided in the leaflet. A positive blood culture from the BacT/ALERT 3D system was used for the study. The BCID2 panel is a multiplexed nucleic acid test designed to detect and identify 33 targets associated with BSIs, including 11 Gram-positive bacteria (*Enterococcus faecalis*, *Enterococcus faecium*, *Listeria monocytogenes*, *Staphylococcus* spp., *Staphylococcus aureus*, *Staphylococcus epidermidis*, *Staphylococcus lugdunensis*, *Streptococcus* spp., *Streptococcus agalactiae*, *Streptococcus pneumoniae*, *Streptococcus pyogenes*), 15 Gram-negative bacteria (*Acinetobacter calcoaceticus-baumannii complex*, *Bacteroides fragilis*, *Enterobacterales*, *Enterobacter cloacae complex*, *Escherichia coli*, *Klebsiella aerogenes*, *Klebsiella oxytoca*, *Klebsiella pneumoniae group*, *Proteus* spp., *Salmonella* spp., *Serratia marcescens*, *Haemophilus influenzae*, *Neisseria meningitidis*, *Pseudomonas aeruginosa*, *Stenotrophomonas maltophilia*) and seven yeast species (*Candida albicans*, *Candida auris*, *Candida glabrata*, *Candida krusei*, *Candida parapsilosis*, *Candida tropicalis*, *Cryptococcus neoformans/gattii*). The BCID2 panel includes tests designed for the targeted identification of various genetic markers associated with resistance to multiple antibiotic classes present in specific Gram-positive (*mecA/C*, *mecA/C*, and MREJ, and *vanA/B*) or Gram-negative bacteria (CTX-M, IMP, KPC, *mcr-1*, NDM, OXA-48, and VIM). Reports on antimicrobial resistance (AMR) genes are only provided if a relevant bacterium is detected. Table 1 summarizes the resistance markers possible to detect using the BICD2 panel, along with the expected resistance phenotype.

### 2.5. Assessment of Therapeutic Decisions

At the 4th Military Clinical Hospital in Wrocław, Poland, there is an antimicrobial stewardship team responsible for hospital antibiotic policy which issued recommendations regarding empirical and targeted therapy for bloodstream infections, based on national recommendations regarding the hospital’s antibiotic list [20], treatment of nosocomial infections [21], and the local epidemiological situation developed from microbiological maps and cumulative antibiograms. Based on these recommendations, a retrospective assessment was made of the correctness of therapeutic decisions made by attending physicians after receiving BCID2 test results. Table 2 details possible BCID2 results, and the preferred therapeutic decisions made depending on the species/resistance marker detected by the molecular test. After obtaining the molecular test result, the therapeutic decisions were considered correct if the treatment applied matched the data in Table 2. Incorrect therapeutic decisions included, for example, failure to de-escalate empirical therapy from carbapenem to third-generation cephalosporin in the case of detecting Gram-negative bacilli without resistance genes, and vice versa in the case of detecting the *bla*_CTX-M_ gene, where the incorrect decision was the failure to escalate therapy to carbapenem. If *Staphylococcus aureus* without the *mecA/C* + MREJ gene has been detected in the BCID2 test, only cloxacillin was considered a valid therapeutic decision, except for patients with a history of penicillin allergy.

### 2.6. Statistics

The Chi-square test or Fisher’s exact test was used to conduct statistical analyses, depending on the fulfillment of assumptions. In cases where the number of categories was large relative to the total number of observations, resulting in many categories having very few cases, no test was applied. Adopting such an approach was due to the risk of insufficient statistical power of each available test to detect differences and the potential impact on the credibility of the analysis results.

### 2.7. Ethics

The study protocol was approved by the Bioethics Committee of the Military Medical Chamber (resolution no. 240/22). The study was carried out according to the guidelines of the Declaration of Helsinki and good clinical practice.

## 3. Results

**Figure 1:**
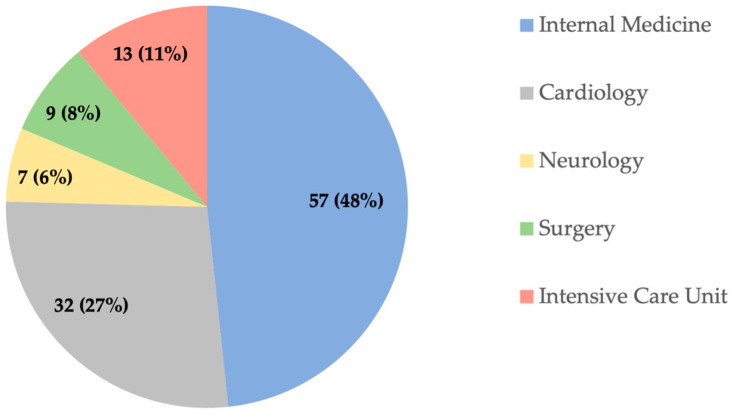
Hospital wards where the examined patients were hospitalized.

A total of 118 patients (45 women, 73 men) with a median age of 76 years were enrolled in the study. Patients were hospitalized in observation, surgical, and intensive care units. The most examined patients were treated in the Department of Internal Medicine (Figure 1).

**Figure 2:**
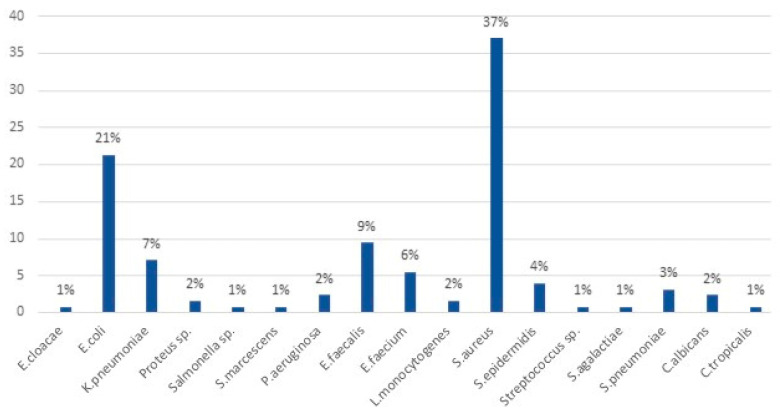
Species structure of isolated microorganisms.

The species structure of isolated microorganisms showed that the most common etiological agents of BSIs in the studied group/population were *Staphylococcus aureus* (37%) and *Escherichia coli* (21%) (Figure 2).

In the case of *S. aureus*, 66% of the strains were sensitive to methicillin (MSSA), while among 47 isolates of Gram (−) bacilli, the ESBL mechanism was detected in 11 strains (26%), three of which also had genes responsible for producing New Delhi metallo-β-lactamases (NDM). All detected antibiotic resistance markers coincided with the phenotypic drugs susceptibility to the cultured microorganisms. Routine culture, conducted in parallel with molecular testing, also showed 100% consistency in the detected microbial species. In detecting *Staphylococcus aureus* in the blood of the examined patient using the molecular method, more than half of the therapeutic decisions made after obtaining the results were incorrect. This percentage was statistically higher (64.5%) if the etiological agent of the BSI was MSSA, compared to 25% of incorrect decisions in detecting the MRSA strain (*p* = 0.01). After receiving the PCR test result, the treatment was generally correct if the etiological factor was Gram (−) bacilli, such as *Escherichia coli* or *Klebsiella pneumoniae* (63 and 67% of correct therapeutic decisions, respectively). In the group where Gram (−) bacilli were the etiological factor of the BSI, statistical significance could not be demonstrated regarding the correctness of therapeutic decision, depending on whether antibiotic resistance genes were detected or not (*p* > 0.05). Bloodstream infections caused by *Enterococcus* spp. accounted for approximately 16% (*Enterococcus faecalis,* 9%; *Enterococcus faecium,* 6%). A more favorable analysis result from the treatment strategy undertaken was demonstrated in the case of the isolation of *E. faecium. Streptococcus pneumoniae* was the etiological factor of the BSI in only four patients. In three patients included in the study, the presence of yeast-like fungi (*Candida albicans*) was detected in the molecular test, so all these patients received antifungal treatment immediately after obtaining the PCR test result. Statistical analysis for Gram (+) microorganisms, other than *Staphylococcus aureus*, was not possible due to a small sample size. The molecular test results have triggered a change in the previously implemented empirical treatment in 54 patients (46%). The antibiotic therapy was corrected after obtaining the final blood culture report, including identifying the microorganism and its drug susceptibility, in 28 patients (24%). Median time from positive blood culture to BCID2 and pathogen identification/susceptibility was 10 h (Q1–Q3, 8–12 h) and 52 h (Q1–Q3, 38–60 h), respectively.

The detailed compilation of detected microorganisms, their resistance mechanisms, and therapeutic decisions is presented in Table 3.

## 4. Discussion

Molecular methods are increasingly becoming an element of routine microbiological diagnostics and have recently revolutionized the approach to managing bloodstream infections. Automation has simplified the PCR procedure, making advanced molecular biology techniques readily available for obtaining standardized results quickly. However, the interpretation of a test performed using the PCR method requires knowledge of the molecular patterns determining the occurrence of bacterial resistance to specific antibiotics [6,9,14,15,22]. Staphylococcal resistance to methicillin is acquired primarily through the *mecA* gene, which encodes a penicillin-binding protein (PBP2a). The *mecA* gene is transferred to the staphylococcal chromosomal cassette mec (SCCmec). The SCCmec cassette integrates into a specific region of the *Staphylococcus* spp. genome, which leads to the formation of MREJ (SCCmec right-end junction). Molecular determination of the area of this junction enables the identification of *S. aureus* MRSA. At the same time, the absence of the *mecA/C* gene will be synonymous with the occurrence of the MSSA phenotype [23,24]. In the case of *S. aureus*-caused BSIs, molecular testing may bring tangible results in the context of faster implementation of targeted antibiotic therapy and earlier abandonment of broad-spectrum empirical treatment.

This is particularly significant due to the high frequency of bloodstream infections (BSIs) caused by *Staphylococcus aureus* in this study. Similar results were obtained in a previous study regarding the etiology of bloodstream infections before and during the COVID-19 pandemic, where *S. aureus* was also the most common etiological agent of BSIs [25]. MSSA accounted for 66% of all detected *Staphylococcus aureus* and was simultaneously the most common etiological factor of BSIs in this study; however, clinicians often made incorrect interpretations of the obtained result and made inappropriate therapeutic decisions when this microorganism was detected. In a situation where, on one hand, we have very strong evidence indicating the presence of MSSA phenotype in the absence of *mecA/C* gene detection and, on the other hand, clear treatment guidelines for infections caused by methicillin-sensitive *Staphylococcus aureus*, the incorrect interpretation of molecular test results seems concerning. This may be due to the need for knowledge of the molecular basis of methicillin resistance in staphylococci. Additionally, it has been shown that it is much more difficult for doctors to decide on the de-escalation of broad-spectrum empirical therapy because of the fear of limiting antibiotic therapy to cloxacillin, i.e., antistaphylococcal penicillin, which should be the treatment of choice in the case of MSSA infections [26]. In cases where the *mecA/C* gene associated with the MRSA phenotype was detected, statistically clinicians less frequently made incorrect therapeutic decisions compared to with MSSA (25% vs. 64.5%; *p* = 0.01). On the one hand, this may be due to a more suggestive molecular test result prompting the physician to suspect an infection caused by a methicillin-resistant strain. On the other hand, it may also be attributed to the ease of making the decision to escalate antibiotic therapy, compared to its de-escalation. Half of the incorrect therapeutic decisions made regarding MSSA in this study were due to continuing empirically-initiated ceftriaxone therapy. In the case of five patients, empirical treatment with vancomycin was continued, and de-escalation to cloxacillin was only performed after receiving the susceptibility test results. A multicenter, retrospective study published in 2023, which analyzed 223 patients with MSSA bacteremia, of which 37 (16.6%) were treated with ceftriaxone, showed that such therapy was associated with a higher risk of treatment failure within 90 days compared to cefazolin or antistaphylococcal penicillin (cloxacillin) [27]. In turn, using glycopeptides to treat infections caused by MSSA is less effective and may even increase mortality [26,28]. The study performed by McDanel et al. showed that in patients with MSSA bloodstream infection, continuation of empirical treatment with vancomycin resulted in increased mortality compared to patients who received targeted beta-lactam antibiotic therapy [29]. The studies conducted by Wong et al. also confirmed these findings and demonstrated that empirical use of vancomycin in suspected *Staphylococcus aureus* infections is appropriate and does not increase mortality, provided that targeted therapy with cloxacillin or cefazolin is initiated within three days of identifying the MSSA strain [30]. Considering the potential nephrotoxicity and reduced effectiveness of vancomycin against MSSA strains, it seems justified to reserve its use for the treatment of infections caused by MRSA [31].

When genes causing resistance to broad-spectrum cephalosporins (ESBL) or carbapenems were detected, optimal changes in antimicrobial treatment were more often made than when a strain without resistance mechanisms was detected. The most common mistake in the case of Gram (−) bacilli was the escalation of empirical therapy, and switching from ceftriaxone to piperacillin with tazobactam or meropenem in the absence of detection of genes responsible for multidrug resistance. This observation also confirms that doctors, in their daily clinical practice, make decisions about escalation rather than de-escalation of antibiotic therapy more easily. Infections of *Enterococcus* spp. etiology are also those in which the species of the detected microorganism strongly determines the appropriate antibiotic therapy. The treatment of choice for *E. faecalis* is ampicillin, while the isolation of *E. faecium* requires vancomycin [32]. In our study, more correct therapeutic decisions were observed if the etiological factor of placental infection was *E. faecium* than *E. faecalis* (71% vs. 58%). As with other microorganisms, the decision to de-escalate therapy to ampicillin was more difficult for the clinician, due to fear of treatment failure.

This analysis indicates a significant need for the possibility of consulting PCR test results within the hospital antimicrobial stewardship. At the same time, it emphasizes that only the correct interpretation of the obtained result can contribute to the limitation of broad-spectrum antibiotic use in therapy, which often causes post-antibiotic complications, such as diarrhea caused by *C. difficile*, and leads to the selection of resistant strains [9]. A study conducted in 2015 by Banerjee et al. showed that measurable benefits from using molecular methods in the diagnosis of BSIs could only be guaranteed by combining them with a hospital antibiotic policy program (ASP). The timing of de-escalation of empiric therapy was strongly dependent on the outcome of the consultation within the ASP. In the group of patients for which antibiotic therapy was intervened, de-escalation occurred on average 20 h (6–36 h) after obtaining a positive blood culture, compared to 39 h (19–56 h) in the control group (without the use of molecular methods), and 36 h (22–61 h) in the group in which the PCR test results were not consulted by the ASP [33].

## 5. Conclusions

In the era of constantly increasing microbial resistance, molecular diagnostic methods enabling earlier implementation of optimal therapeutic decisions should be used, in parallel with classic cultures, followed by identification and antibiogram of the microorganism constituting the etiological factor of the infection. Considering the frequency of bloodstream infections (BSIs) caused by *Staphylococcus aureus*, the 100% concordance of molecular test results with routine microbial culture, and the results of this observation, the conclusion should be drawn that only correct and conscious interpretation of molecular tests can have a measurable impact on the protection of antibiotics and the improvement of treatment effects. Due to the high rate of incorrect interpretations of molecular test results in this study, it seems that only consultation within an antimicrobial stewardship program enables full use of the potential of these tests. Additionally, it is necessary to continuously educate medical staff on the mechanisms that determine bacterial resistance to antibiotics and the principles of their rational use.

## Figures and Tables

**Table 1:** Resistance markers possible to detect with the BCID2 test.

**Gram-Positive Resistance Markers**	
**Gene**	**Resistance Phenotype**
*vanA/B*	Marker for vancomycin-resistant Enterococcus (VRE).
*mecA/C* *mecA/C* + MREJ	*mecA/C* is a marker for methicillin resistance in non-*S. aureus Staphylococci* (MRCoNS) but reported only in *Staphylococcus epidermidis* and *Staphylococcus lugdunensis.* MREJ is only evaluated in *Staphylococcus aureus* and when detected with *mecA/C,* is specific for MRSA.
**Gram-Negative Resistance Markers**	
**Gene**	**Resistance Phenotype**
CTX-M (*bla*_CTX-M_)	The marker for the most common extended spectrum β-lactamase (ESBL) frequently identified in gram-negative pathogens, especially *Escherichia coli* and *Klebsiella* spp., is outlined. ESBLs are enzymes capable of hydrolyzing extended-spectrum cephalosporins (e.g., ceftriaxone, cefepime) and piperacillin/tazobactam. It is important to note that a negative result does not necessarily exclude the presence of other ESBL enzymes or alternative beta-lactamases.
KPC (*bla*_KPC_) NDM (*bla*_NDM_), VIM (*bla*_VIM_), IMP (*bla*_IMP_) OXA-48 like (*bla*_OXA-48-like_)	Markers for carbapenemases producing Gram-negative bacilli.
*mcr-1*	Marker for colistin resistance

**Table 2:** Preferred therapeutic decisions based on possible BCID2 test results.

Microorganism	Possible BCID2 Result		Preferred Therapy
*Enterococcus faecalis*	*vanA/B*	not detected	ampicillin
		detected	ampicillin
*Enterococcus faecium*	*vanA/B*	not detected	vancomycin
		detected	linezolide
*Staphylococcus aureus*	*mecA/C* + MREJ	not detected	cloxacillin
		detected	vancomycin
CoNS	*mecA/C*	not detected	cloxacillin
		detected	vancomycin
*Streptococcus pneumoniae*	-		III generation cephalosporin
*Listeria monocytogenes*	-		ampicillin
*Streptococcus pyogenes*	-		penicillin
*Streptococcus agalactiae*	-		penicillin
*Enterobacterales orders only* or *Enterobacter cloacae complex* *Klebsiella (Enterobacter) aerogenes* *Serratia marcescens*	CTX-M	not detected	cefepime
		detected	meropenem
	KPC	detected	ceftazidime/avibactam
	IMP	detected	colistin + aminoglycoside
	NDM	detected	
	VIM	detected	
	OXA-48	detected	ceftazidime/avibactam
*Escherichia coli* *Klebsiella pneumoniae group* *Klebsiella oxytoca* *Proteus* spp. *Salmonella* spp.	CTX-M	not detected	III generation cephalosporin
		detected	meropenem
	KPC	detected	ceftazidime/avibactam
	IMP	detected	colistin + aminoglycoside
	NDM	detected	
	VIM	detected	
	OXA-48	detected	ceftazidime/avibactam
*Acinetobacter baumannii*	-		colistin + meropenem
*Pseudomonas aeruginosa*	-		ceftazidime
*Stenotrophomonas malthophilia*	-		trimethoprim/sulfamethoxazole
*Bacteroides fragilis*	-		metronidazole
*Haemophilus influenzae*	-		III generation cephalosporin
*Neisseria meningitidis*	-		III generation cephalosporin
*Candida* spp.	-		echinocandin alternative: fluconazole as an initial therapy in selected patients who are not critically ill, and who are considered unlikely to have a fluconazole-resistant *Candida* species
*Cryptococcus neoformans*	-		amphotericin B, fluconazole

CoNS—coagulase-negative staphylococci.

**Table 3:** Analysis of isolated microorganisms and therapeutic decisions made after obtaining the molecular test result. A. Change of empiric therapy after obtaining molecular test results. B. Change of empiric therapy after obtaining the culture results.

	Number of Cases *n*	A *n* (%)	B *n* (%)	Therapeutic Decision after Obtaining Molecular Test Results		*p* Value
				Correct *n* (%)	Incorrect *n* (%)	
***S. aureus***	47	23(49)	16(34)	23(49)	24(51)	-
MSSA	31	15(48)	14(45)	11(35.5)	20(64.5)	0.01
MRSA	16	11(69)	2(12,5)	12(75)	4(25)	
***E. coli***	27	6(22)	2(7)	17(63)	10(37)	-
CTX-M (+)	3	1(33)	1(33)	2(67)	1(33)	0.88
No resistance gene detected	24	5(21)	1(4)	15(62.5)	9(37.5)	
***K. pneumoniae***	9	4(44)	1(11)	6(67)	3(33)	-
CTX-M (+)	4	2(50)	1(25)	3(75)	1(25)	0.83
CTX-M (+) NDM (+)	3	1(33)	0	2(67)	1(33)	
No resistance gene detected	2	1(50)	0	1(50)	1(50)	
***P. aeruginosa***	3	2(67)	0	0	3(100)	-
***Proteus* spp.**	2	1(50)	0	1(50)	1(50)	-
***Salmonella* spp.**	1	0	0	1(100)	0	-
***S. marcescens***	1	1(100)	0	0	1(100)	-
**Gram (−) bacilli**	43	37(86)	19(44)	25(58)	18(42)	-
CTX-M (+)	7	3(43)	2(29)	5(71)	2(29)	0.68
CTX-M (+) NDM (+)	3	1(33)	0	2(67)	1(33)	
No resistance gene detected	33	10(30)	1(3)	18(55)	15(45)	
***E. faecalis***	12	9(75)	5(42)	7(58)	5(42)	-
***E. faecium***	7	5(71)	4(57)	5(71)	2(29)	-
***S. epidermidis***	5	4(80)	1(20)	3(60)	2(40)	-
MRCNS	5					
***S. pneumoniae***	4	0	1(25)	3(75)	1(25)	-
***C. albicans***	3	3(100)	1(33)	3(100)	0	-
***L. monocytogenes***	2	1(50)	0	1(50)	1(50)	-
***S. agalactiae***	1	0	0	0	1(100)	-
***Streptococcus* spp.**	1	0	0	1(100)	0	-

MSSA, methicillin-sensitive *Staphylococcus aureus*; MRSA, methicillin-resistance *Staphylococcus aureus*; CTX-M, extended-spectrum β-lactamases (ESBL); NDM, New Delhi metallo-β-lactamases; MRCNS, methicillin-resistant coagulase-negative staphylococci.

## Data Availability

Derived data supporting the findings of this study are available from the first author Natalia Słabisz upon request.
